# Amide proton transfer weighted (APTw) imaging based radiomics allows for the differentiation of gliomas from metastases

**DOI:** 10.1038/s41598-021-85168-8

**Published:** 2021-03-09

**Authors:** Elisabeth Sartoretti, Thomas Sartoretti, Michael Wyss, Carolin Reischauer, Luuk van Smoorenburg, Christoph A. Binkert, Sabine Sartoretti-Schefer, Manoj Mannil

**Affiliations:** 1 Institute of Radiology, Kantonsspital Winterthur, Winterthur, Switzerland; 2 Faculty of Medicine, University of Zürich, Zürich, Switzerland; 3 Philips Healthsystems, Zürich, Switzerland; 4 Department of Medicine, University of Fribourg, Fribourg, Switzerland; 5 Department of Radiology, HFR Fribourg-Hôpital Cantonal, Fribourg, Switzerland; 6 Department of Neuroradiology, Kantonsspital Aarau, Aarau, Switzerland; 7 Institute of Clinical Radiology, University Hospital Münster, University of Münster, Albrecht-Schweitzer-Campus 1, E48149 Münster, Germany

**Keywords:** Biomarkers, Health care, Medical research, Neurology, Oncology, Signs and symptoms

## Abstract

We sought to evaluate the utility of radiomics for Amide Proton Transfer weighted (APTw) imaging by assessing its value in differentiating brain metastases from high- and low grade glial brain tumors. We retrospectively identified 48 treatment-naïve patients (10 WHO grade 2, 1 WHO grade 3, 10 WHO grade 4 primary glial brain tumors and 27 metastases) with either primary glial brain tumors or metastases who had undergone APTw MR imaging. After image analysis with radiomics feature extraction and post-processing, machine learning algorithms (multilayer perceptron machine learning algorithm; random forest classifier) with stratified tenfold cross validation were trained on features and were used to differentiate the brain neoplasms. The multilayer perceptron achieved an AUC of 0.836 (receiver operating characteristic curve) in differentiating primary glial brain tumors from metastases. The random forest classifier achieved an AUC of 0.868 in differentiating WHO grade 4 from WHO grade 2/3 primary glial brain tumors. For the differentiation of WHO grade 4 tumors from grade 2/3 tumors and metastases an average AUC of 0.797 was achieved. Our results indicate that the use of radiomics for APTw imaging is feasible and the differentiation of primary glial brain tumors from metastases is achievable with a high degree of accuracy.

## Introduction

Amide proton transfer weighted (APTw) imaging represents a novel contrast media free molecular MR imaging technique that has recently shown promise in characterizing and differentiating brain neoplasms as well as malignancies in other body regions^1–7^. The APTw signal originates from amide protons in endogeneous proteins and peptides in the parenchyma. In tumor tissue, the content of mobile proteins and peptides is increased thus resulting in increased APTw signal intensity values^8,9^.

With the exception of one study^10^, these previous investigations utilized standard histogram analyses techniques at the most to analyse the APTw signal of the tissue at hand thus only scratching the surface of the information that can potentially be extracted from radiological images^1–3^.

With recent advances in the field of machine learning (ML), radiomics techniques allowing for the extraction of high-dimensional mineable data from medical images have been developed and introduced to medical imaging thus enabling in-depth tissue classification and characterization^11–15^.

In this proof-of-concept study we aimed at assessing the potential of radiomics and ML for APTw imaging. To this extent, we used radiomics on APTw images to differentiate WHO Grade 2, 3 and 4 gliomas from brain metastases.

## Materials and methods

This study received institutional review board approval (Cantonal Ethical Committee Zürich, Switzerland) and was performed in accordance with all guidelines and regulations defined by the institutional review board. All subjects gave written informed general consent.

### Subjects

In this retrospective study we included 48 patients (mean age: 61 years, range: 37–83 years) diagnosed with either low- or high grade gliomas (10 patients with WHO Grade 2 tumors, 1 patient with WHO Grade 3 tumor and 10 patients with WHO Grade 4 tumors (= glioblastoma)) or brain metastases (MET; 27 patients, with 15 lung, 1 breast, 9 melanoma and 2 kidney as primary sites of origin). Initially 64 consecutive patient studies acquired between august 2018 and april 2020 at a single tertiary institution were reviewed. Then the following exclusion criteria were applied: < 18 years, lack of histological analysis of neoplasm (6 patients), unavailable MRI data (1 patient), treatment prior to MRI, lesions with a diameter of under 10 mm^16^ (3 patients), failure to compute APTw signal intensities during image reconstruction (6 patients). In the case of multiple metastases all lesions were used for analysis (if they fulfilled size requirements) and were pooled^17^ thus resulting in one data point per patient. A flowchart can be found in the *supplementary material*.

### MR imaging

Subjects were examined on a clinical 3 T scanner (Achieva, Philips Healthcare, Best, the Netherlands) with an eight channel receive-only head coil array. The following sequences were acquired: precontrast transverse diffusion-weighted imaging (DWI), 3D double inversion recovery (DIR), 3D T1-weighted (T1w) turbo field echo (TFE) and postcontrast 3D fluid attenuated inversion recovery (FLAIR), 3D T1 black blood turbo spin echo (TSE) or 3D T1w m-Dixon TFE, APTw and 2D T2w TSE.

Table 1 shows the sequence parameters for the APTw sequence.

**Table 1:** APTw MRI sequence parameters.

	3D APTw sequence
FOV	228 × 178 × 60 mm^3^
Scan mode; Acquisition voxel ; Reconstruction voxel	3D; 1.8 × 1.8 × 6.0 mm^3^; 0.9 × 0.9 × 3.85 mm^3^
Reconstruction matrix	256 × 256
Slice thickness, Slice gap	3.85 mm, 0 mm
SENSE or Compressed SENSE factor	1.6 SENSE
TSE factor	174
Rest slabs	0
Flip angle (in degrees)	90
TR, TE and TE equivalent	TR 5800 to 5864 ms; TE 7.8 to 8.3 ms
Inversion time TI	–
Fat suppression	SPIR
APTw	saturation B_1 rms_: 2 µT; saturation duration: 2 s
Number of acquisitions NSA	1
Scan duration	03 min 42 s

We used a clinically approved APTw sequence^18–20^ acquired in transverse oblique orientation parallel to the intercommissural line. 16 slices with a slice thickness of 3.85 mm were acquired. The first slice was centered at the inferior border connecting the rostrum and the splenium of the corpus callosum^19,20^. Details on how APTw imaging contrast was generated can be found in the *supplementary material*.

### Radiomics feature extraction and image analysis

**Figure 1:**
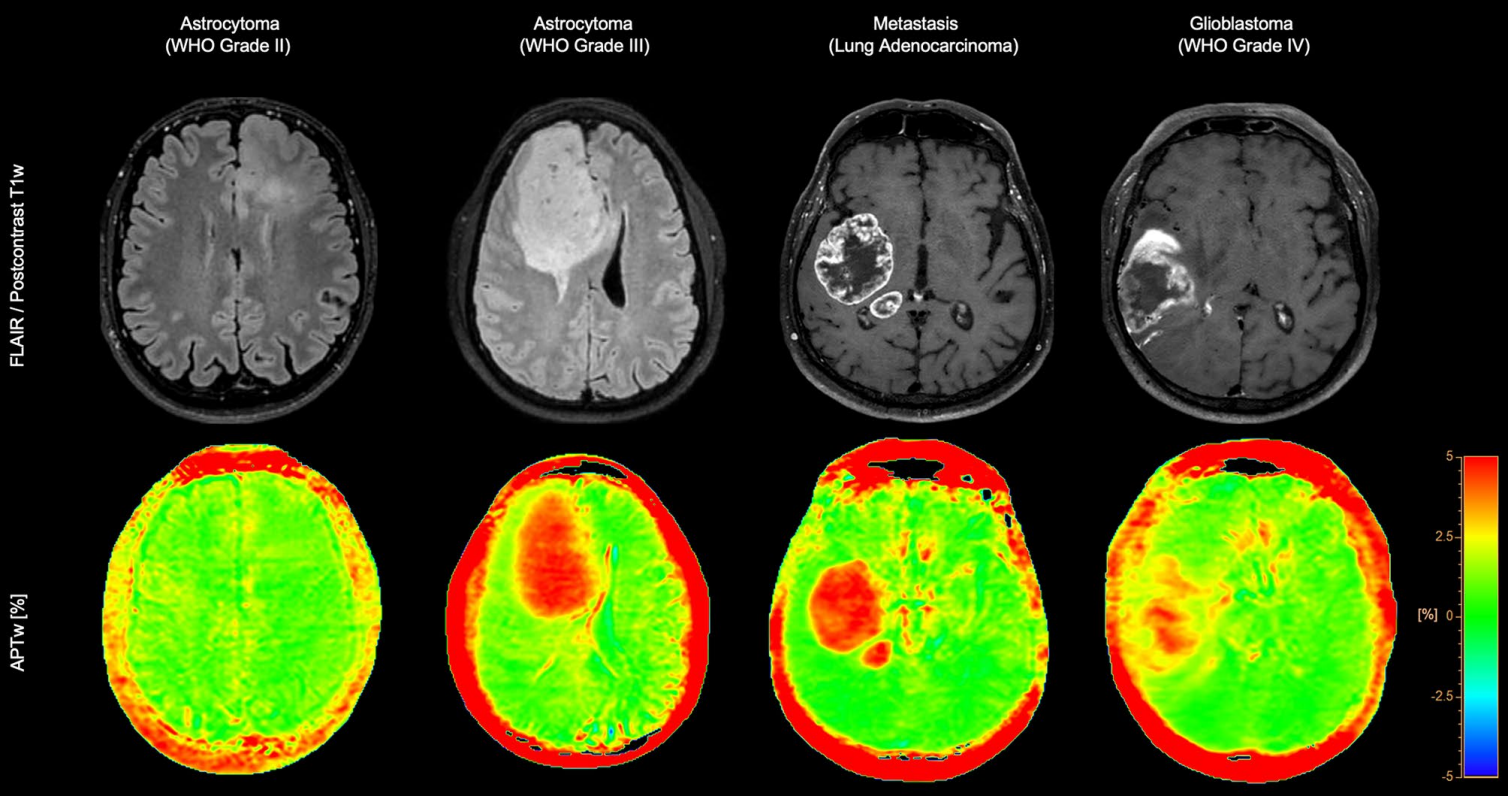
Representative image examples of 4 patients who presented with different neoplasms. FLAIR or T1w postcontrast images are provided in the top row, while the corresponding APTw images are shown in the bottom row.

APTw and postcontrast T1w or FLAIR Digital Imaging and Communications in Medicine (DICOM) files were loaded into the open-source software platform 3D Slicer (v. 4.10.2) and were aligned geometrically. Subsequently, two readers (TS and ES each with 3 years of experience) manually segmented the neoplasms on overlayed images according to contrast enhancement and solid parts of neoplasms^21^. Segmentation was performed on all axial slices for 3D segmentation of either T1w postcontrast or FLAIR images superficially overlaid onto the APTw images (Fig. 1). Specifically, readers outlined neoplasms on pseudo-images that were generated by accurately overlaying APTw with the now geometrical identical structural images. These pseudo-images contain the APTw imaging information but visually reflect T1w postcontrast or FLAIR images. Intensity discretization was performed to a bin width of 25. Gray level cooccurrence matrix (GLCM) features were computed at 4 inter-pixel distances.

Then 110 radiomics features were extracted with the built-in pyRadiomics package implemented into 3D Slicer^22^. Most features are in accordance with those described in the Imaging Biomarker Standardization Initiative (IBSI)^23^. Radiomics features corresponded to seven different matrices/feature classes: First-order statistics/histogram matrix, shape-based features, gray-level cooccurrence matrix (GLCM), gray-level run length matrix (GLRLM), gray-level size zone matrix (GLSZM), neighbouring gray tone difference matrix (NGTDM), and gray-level dependence matrix (GLDM). A detailed overview and description of radiomics features can be found elsewhere^22,24^.

### Radiomics features—dimension reduction

Dimension reduction was performed in two steps. First, radiomics features of both readers were compared by means of intraclass correlation coefficients (ICC). ICC values of greater than 0.8 were interpreted as excellent agreement^11,25^. Radiomics features with ICC values below this threshold were discarded from further analysis, as shown previously^26^. In a second step, a classifier attribute evaluation filter (CfsSubsetEval) of the open source software package Weka (WEKA, version 3.8.3, University of Waikato, Hamilton, New Zealand) were applied on the training data to evaluate the worth of an attribute. This method measures the significance of attributes on the basis of predictive ability of attributes and its degree of redundancy. The subsets which are having less intercorrelation but are highly correlated to the target class are selected for further analyses.

The remaining radiomics features were then used to train ML classifiers. Combinations of the weighted radiomics features were used then to distinguish metastases from glial primary brain tumours.

For the consecutive subanalyses of distinguishing glioblastomas from other glial brain tumors and metastases, we performed a prior principal component analysis to cover approximately 95% of variance in the original dataset.

### Machine learning

For ML analysis, open-source software (WEKA, version 3.8.3, University of Waikato, Hamilton, New Zealand) was used. For prediction of histopathology, a commonly-used ML algorithm implemented in the open source WEKA package was tested with handpicked hyperparameters: Multilayer perceptron, which uses backpropagation to learn a multi-layer perceptron to classify instances with a learning rate of 0.3 and a momentum of 0.2. For further subanalyses we used a random forest classifier. All results were tenfold cross validated to overcome overfitting.

## Results

### Dimension reduction

After dimension reduction regarding reproducibility and attribute evaluation, eight out of 110 radiomics features remained for further analysis (Table 2).

**Table 2:** Radiomics features after dimension reduction.

#	Radiomics features
1	Voxel volume
2	Mesh volume
3	Dependence Non uniformity normalized
4	Large dependence high gray level emphasis
5	Low gray level emphasis
6	Sum average
7	Zone variance
8	Zone percentage

### Machine learning

**Figure 2:**
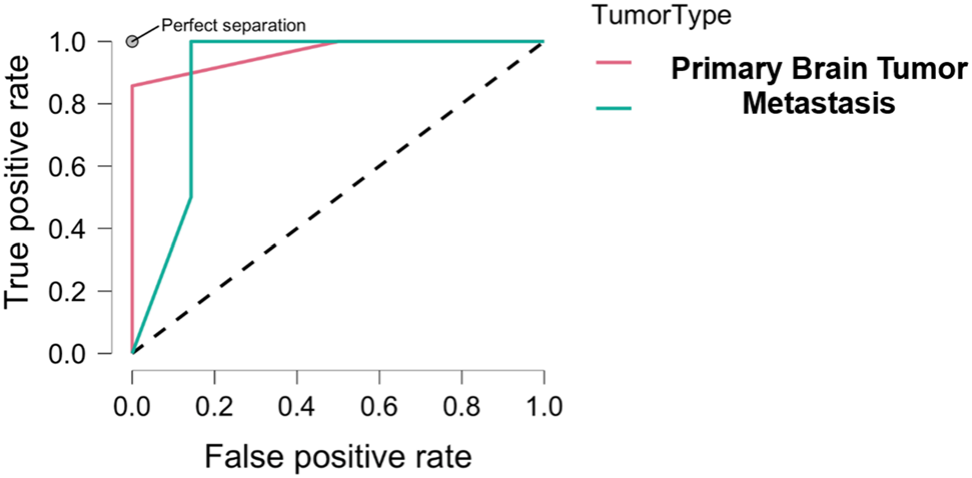
Receiver-operating-characteristics (ROC) of the machine learning algorithm to correctly identify primary brain tumors and metastases. The overall area-under-the-curve (AUC) was 0.836.

The Multilayer Perceptron classifier yielded a sensitivity of 81.3%, a specificity of 81.1%, a recall of 0.81, F-measure 0.81, and an area under the curve (AUC) in receiver operating characteristics (ROC) of 0.836 (Fig. 2) in distinguishing primary brain tumors (glial tumors and glioblastomas) from metastases.

### Subanalysis I

In the subanalysis of primary brain tumors, the random forest classifier was able to distinguish glioblastomas from other glial cell tumors (WHO I-III) with a sensitivity of 90.5%, a specificity of 90.4%, a recall of 0.905, F-measure 0.905, and an area under the curve in receiver operating characteristics of 0.868.

### Subanalysis II

**Figure 3:**
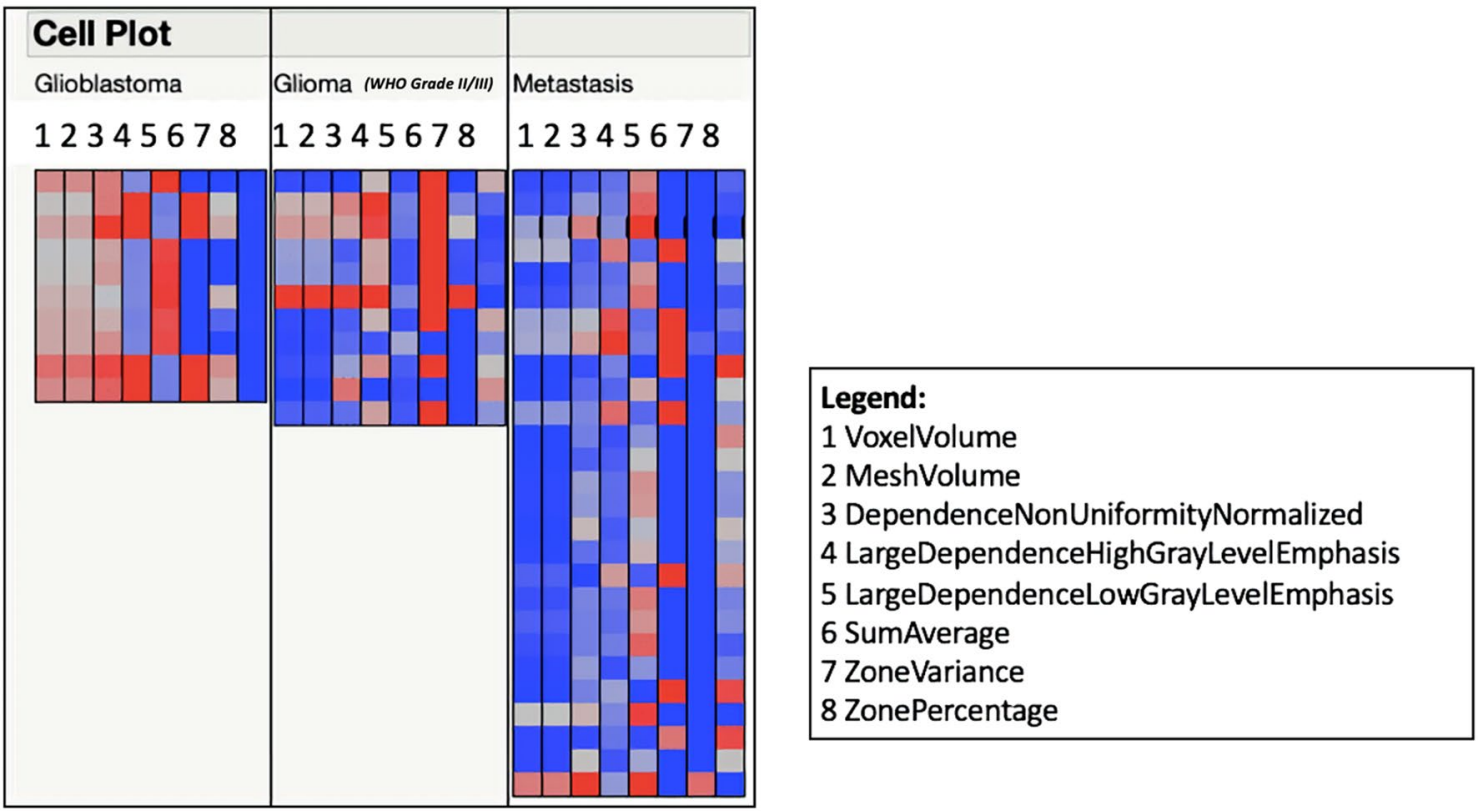
Cell plot of eight standardized Radiomics features visualize similarities between glioblastomas, other gliomas and metastases. Stark differences can be observed in the values of glioblastomas and metastases.

**Figure 4:**
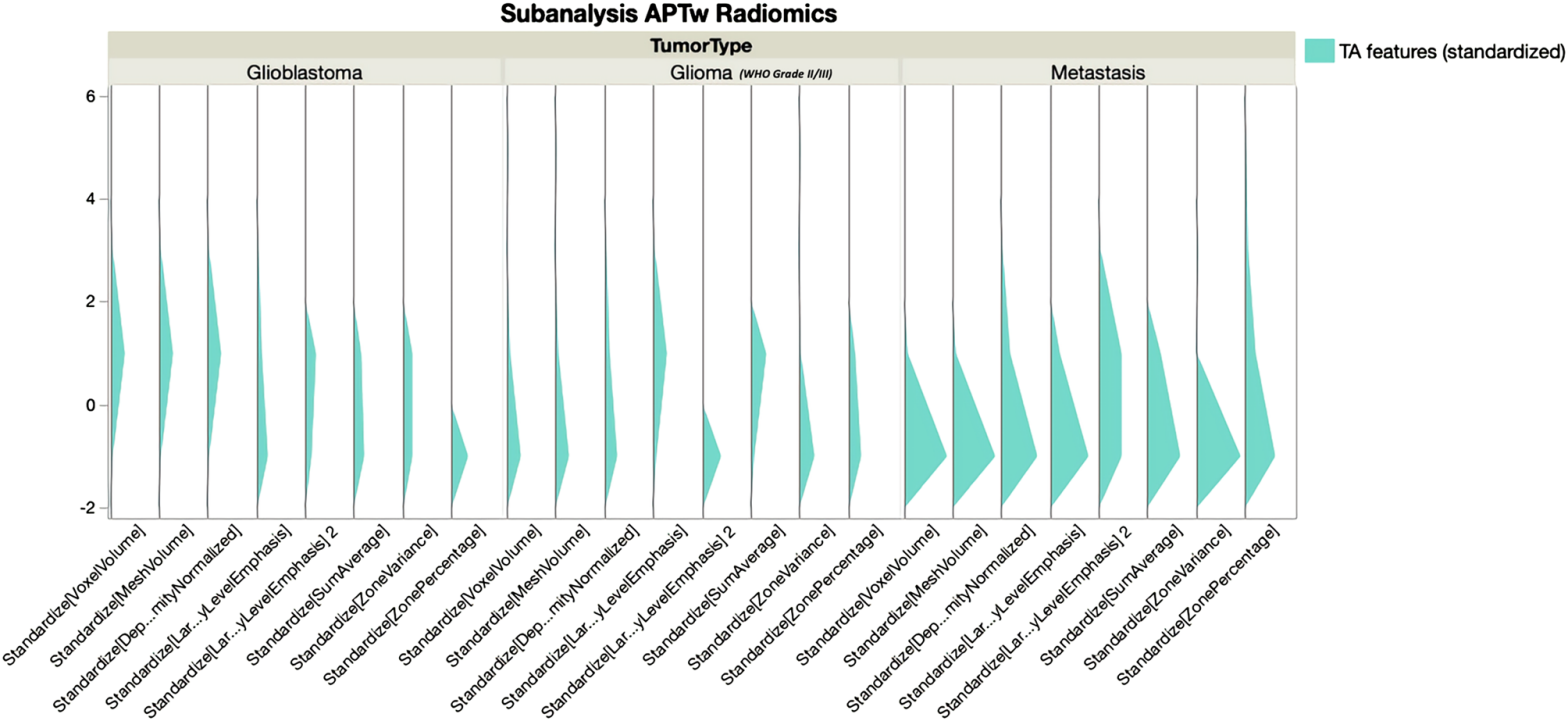
Relative visualization of the eight standardized texture analysis features for glioblastomas, other gliomas and metastases. Overall lower values are observed for metastases compared to glioblastomas and other gliomas, respectively.

In the subanalysis of primary brain tumors combined with metastases (Figs. 3, 4), the random forest classifier was able to distinguish these entities with an average sensitivity of 62.5%, a specificity of 74.9%, a recall of 0.625, F-measure 0.628, and an area under the curve in receiver operating characteristics of 0.797 after stratified tenfold cross validation.

## Discussion

In this proof-of-concept study we assessed the utility of radiomics for APTw imaging. To this end, we used radiomics features and machine learning algorithms to differentiate glioblastomas from gliomas and brain metastases. Our results indicate that the application of radiomics to APTw imaging is feasible and allows for the differentiation of these brain neoplasms.

APTw is a novel molecular MRI technique that relies on endogenous cellular proteins in vivo to generate contrast. APTw imaging belongs to the chemical exchange saturation transfer (CEST) imaging group and is the only form of CEST imaging that has yet achieved FDA approval.

The APTw signal is theoretically caused by two major sources: Firstly, the intracellular water-exchangeable amide proton content in the cytoplasm and secondly the base-catalyzed exchange rate at physiological pH range^27^. Incidentally however, the APTw signal is not pure and may be contaminated by a variety of sources^28–33^. Specifically, the water longitudinal relaxation time (T1) may influence the APTw signal. T1 effects (T1 recovery and T1 related saturation) may influence the APTw signal linearly or in a more complex manner depending on the level of direct water saturation effects, the field strengths of the MR scanner, irradiation power and whether non-steady-state or steady-state acquisitions are performed. Furthermore. The APTw signal may also be affected by semi-solid magnetization-transfer (MT) effects and other nearby CEST and relayed nuclear Overhauser enhancement (rNOE) saturation transfer effects. Ultimately, APTw intensity values may also be impacted by B1 effects which can be triggered by an imperfect distribution of the irradiation power across the brain.

Currently, APTw is mainly used for brain tumor imaging. With malignant brain tumors exhibiting a high degree of protein content, the APTw signal increases steadily with the amount of protein content relative to the surrounding parenchyma. This has been successfully leveraged for differentiating and grading tumors according to their WHO grade^34^. Specifically, a recent meta-analysis listed the sensitivity and specificity of APTw for differentiating high grade from low-grade glial tumors as 88% and 91% respectively^35^. Furthermore, based on this principle, high grade regions can be identified within histologically heterogenous brain tumors, thus allowing for more accurate sampling during stereotactic biopsies. Additionally, APTw has also been successfully employed for monitoring tumor response to therapies such as radio-/chemotherapy or high-intensity focused ultrasound. Most importantly, APTw enables the differentiation of recurrent tumor from treatment effects such as radiation necrosis. Lastly, APTw has also been successfully used for identifying genetic markers in gliomas, such as the MGMT or IDH status^34^.

While APTw has already proven to be a valuable addition to the field of tumor imaging, the development of innovative approaches to further leverage the potential of APTw is highly desirable. Radiomics has become a popular method for extracting more data from radiological images thus enabling in-depth study of the tissue at hand^12,36^. Therein numerous studies have shown that radiomics approaches based on various imaging modalities (i.e. T1w, T2w, FLAIR, DWI, ADC, SWI, DTI) enhance brain tumor imaging^12,16,21^.

Here we further enhance the spectrum of radiomics in terms of 3D texture analysis (TA) for brain tumor imaging by applying it to APTw imaging. Our approach yielded a high degree of accuracy in differentiating different types of brain tumors. After dimension reduction all first order TA features were excluded from further analyses due to high intercorrelation. A total of eight features remained for further analysis:

(1) *Dependence Non-Uniformity Normalized* (DNN), which measures the similarity of voxel dependence throughout the image, an indirect measure of homogeneity. (2) *LargeDependenceHighGrayLevelEmphasis (LDHGLE)*, which measures the joint distribution of large dependence of higher gray-level values. (3) *Low Gray Level Emphasis (LGLE)*, which measures the distribution of low gray-level values, and (4) *SumAverage*, which measures the relationship between occurences of pairs with lower intensity values and occurences of pairs with higher intensity values. All four aforementioned features are derived from the *GrayLevelDependenceMatrix (GLDM)*, which are used to quantify gray level dependencies in a medical image by taking into account neighbouring voxels. The remaining features were associated with the size of the neoplasms. While *VoxelVolume* and *MeshVolume* are directly correlated with size, *ZoneVariance* measures the variance in zone size volumes. Similarly, *ZonePercentage* takes into account the number of zones and number of voxels within a defined region of interest, which represents the coarseness of texture. While gliomas were found to exhibit a larger overall size than metastases, which was reflected in these features, they may have no true biological value as metastases and gliomas can present with various sizes.

A major limitation of the current study is its retrospective, single centre design and its small sample size with heterogeneous spectrum of pathologies. It should however be noted, that due to the novelty of the imaging modality (i.e. clinical approval of the sequence occurred in 2018) a larger dataset is not available at the time. Furthermore, larger datasets are likely to decrease the risk of overfitting the machine learning classifiers. We counteracted these limitations by implementing tenfold cross validation of our results. Moreover, the quality of segmentations may strongly influence radiomics results^37^. Two readers segmented the neoplasms and subsequently features with low interreader agreement were excluded thus reducing bias. However, we acknowledge that there may be more sophisticated methods of performing segmentations and of reducing reader bias. Lastly, our study was performed on a single MR scanner, which limits the generalizability of our findings. The influence of different acquisition parameters, sequences, field strengths, and MR scanners on 3D TA features in APTw images remains to be investigated.

In conclusion, we show that radiomics allows for the differentiation of various brain neoplasms on APTw images. The current work justifies the further study and development of radiomics for APTw imaging in an effort to widen the applicability and utility of APTw imaging for various diseases and anatomies.

## Supplementary Information

Supplementary Information

## Data Availability

Data can be made available upon reasonable request to the corresponding author.

## Abbreviations

APTw: Amide Proton Transfer weighted
ML: Machine learning
TA: Texture analysis
GLRLM: Gray-level run length matrix
GLSZM: Gray-level size zone matrix
NGTDM: Neighbouring gray tone difference matrix
GLDM: Gray-level dependence matrix
CEST: Chemical Exchange Saturation Transfer
