# Supplementary material for: Amide proton transfer weighted (APTw) imaging based radiomics allows for the differentiation of gliomas from metastases
Source: Sci Rep. 2021 Mar 9;11:5506. doi: 10.1038/s41598-021-85168-8 (PMC7943598; doi:10.1038/s41598-021-85168-8)
Supplement: Supplementary file 1 — Supplementary Information [file 41598_2021_85168_MOESM1_ESM.docx]

**Amide Proton Transfer weighted (APTw) Imaging based Radiomics allows for the Differentiation of Gliomas from Metastases**

Elisabeth Sartoretti, Thomas Sartoretti, Michael Wyss, Carolin Reischauer, Luuk van Smoorenburg, Christoph A. Binkert, Sabine Sartoretti-Schefer, Manoj Mannil

**Supplementary Material**

**Figure 1s**: Flowchart

**
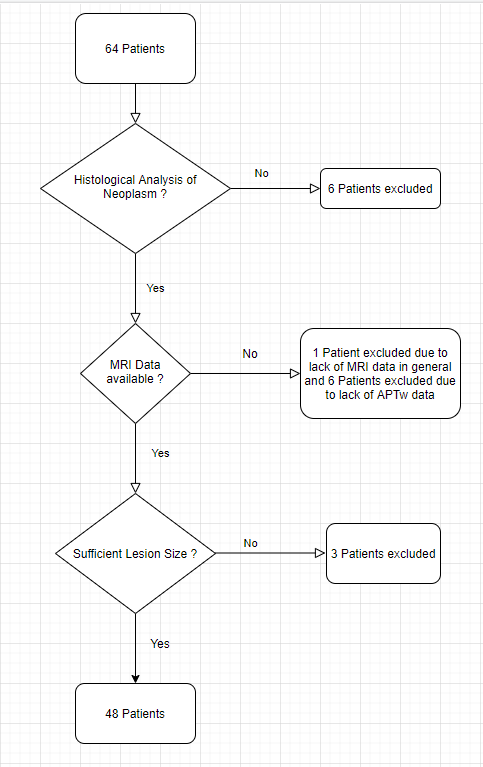
**

APTw sequence and APTw Image Contrast

To generate APTw imaging contrast, magnetization transfer ratio asymmetry (MTR_asym_) was calculated according to the following formula:

$${MTR}_{asym} \left( \% \right)= \frac{(S_{-\Deltaɷ}-S_{\Deltaɷ})}{S_{0}}$$

S_-∆ɷ_ and S_∆ɷ_ correspond to the water signal at negative and positive frequency offset. S_0_ is the signal without radiofrequency saturation. MTR_asym_ is based on the acquisition of a Z-spectrum, where multiple water signal levels are measured as a function of different frequency offsets (∆ɷ). The water signal saturation is calculated as a function of the saturation frequency on this spectrum (1,2,3).

For the Z-spectrum, nine image volumes at seven different frequency offsets (± 3.1 ppm, ± 3.5 ppm, ± 3.9 ppm, and −1560 ppm) were acquired (1,2,3). A B_0_ map derived from three acquisitions at +3.5 ppm with slightly different echo shifts using an mDIXON algorithm was used for a voxel-by-voxel B_0_ correction (1,2,3). B_1_ shimming was performed for each scan thus allowing for B_1_ inhomogeneity correction as described in detail by Togao et al. (1). APTw intensity values in this paper always represent MTR_asym_ values at 3.5 ppm offset frequency (∆ɷ) quantified in % water signal intensity (1,2,3), as seen in the following formula:

$$APTw (\%)= {MTR}_{asym}[\Deltaɷ= +3.5ppm](\%)$$

**References**

*1. Togao O, Hiwatashi A, Keupp J, Yamashita K, Kikuchi K, Yoshiura T, Suzuki Y, Kruiskamp MJ, Sagiyama K, Takahashi M, Honda H. Scan-rescan reproducibility of parallel transmission based amide proton transfer imaging of brain tumors. J Magn Reson Imaging 2015;42(5):1346-1353. doi: 10.1002/jmri.24895*

*2. Sartoretti T, Sartoretti E, Wyss M, Schwenk Á, Najafi A, Binkert C, Reischauer C, Zhou J, Jiang S, Becker AS, Sartoretti-Schefer S. Amide Proton Transfer Contrast Distribution in Different Brain Regions in Young Healthy Subjects. Front Neurosci 2019;13:520. doi: 10.3389/fnins.2019.00520*

*3. Sartoretti E, Sartoretti T, Wyss M, Becker AS, Schwenk Á, van Smoorenburg L, Najafi A, Binkert C, Thoeny HC, Zhou J, Jiang S, Graf N, Czell D, Sartoretti-Schefer S, Reischauer C. Amide Proton Transfer Weighted Imaging Shows Differences in Multiple Sclerosis Lesions and White Matter Hyperintensities of Presumed Vascular Origin. Front Neurol 2019;10:1307. doi: 10.3389/fneur.2019.01307*
